# Supplementary material for: Template-Assisted Assembly of Hybrid DNA/RNA Nanostructures Using Branched Oligodeoxy- and Oligoribonucleotides
Source: Int J Mol Sci. 2023 Nov 5;24(21):15978. doi: 10.3390/ijms242115978 (PMC10650595; doi:10.3390/ijms242115978)
Supplement: Supplementary file 1 [file ijms-24-15978-s001.zip › ijms-2560691-supplementary.pdf]

Supplementary Material

Template-Assisted Assembly of Hybrid DNA/RNA Nanostructures from Branched Oligodeoxy- and Oligoribonucleotides

Alesya Fokina[1,2#], Yulia Poletaeva[3#], Svetlana Dukova[4], Kristina Klabenkova[1,2], Zinaida Rad′kova[1], Anastasia Bakulina[1], Timofei Zatsepin[5], Elena Ryabchikova[3], and Dmitry Stetsenko[1,2*]

[1] Faculty of Physics (A.F. and K.K.) and Faculty of Natural Sciences (Z.R. and A.B.), Novosibirsk State University, Novosibirsk 630090, Russia; a.fokina@nsu.ru (A.F.), k.klabenkova@g.nsu.ru (K.K.), 79513891932@yandex.ru (Z.R.), bakulina@gmail.com (A.B.)
[2] Institute of Cytology and Genetics, Siberian Branch of the Russian Academy of Sciences, Novosibirsk 630090, Russia; stetsenkoda@bionet.nsc.ru (D.S.)
[3] Institute of Chemical Biology and Fundamental Medicine, Siberian Branch of the Russian Academy of Sciences, Novosibirsk 630090, Russia; fabaceae@yandex.ru (Yu.P.), lenryab@niboch.nsc.ru (E.R.)
[4] JSC Genterra, Moscow 129085, Russia; kurakina_svetlan@mail.ru (S.D.)
[5] Department of Chemistry, Lomonosov Moscow State University, Moscow 119991, Russia; tsz@yandex.ru (T.Z.)
[*] Correspondence: stetsenkoda@bionet.nsc.ru, tel.: +7-383-363-4963.
[#] The authors who made equal contributions to this work.

Table S1. Structures and molecular masses of the branched oligonucleotides obtained.

Designation	Sequence	Molecular mass, Da [1]	
		Calc.	Exper.
**A**	(5′-a*g*tctcgacttgctacctcat-3′-)$_2$-Y-3′-<br>UACU*UAAGUAGUGUCCGAUUCA*C*A-5′	20805.6	20803.6
**B**	5′-(U*U*CAUCACAGGCUAAGUGU*tt-3′-)$_2$-Y-3′-<br>UACU*UAAAAUCACGACUAUGUG*U*U-5′	21783.6	21777.9
**C**	5′-(U*U*UUAGUGCUGAUACACAA*tt-3′-)$_3$-Ψ-5′-<br>CAUCGC***t***GCGCAG*C*G-3′	18498.6	18494.6
**F**	5′-(t*a*ta***t***atataa***t***attata***t***atatatct-3′-)$_3$-Ψ-O-$(CH_2)_6$-S-S-$(CH_2)_6$-<br>Op-3′-UCAGAGCUGAACGAUGGAG*U*A-5′	33381.6	33372.9
**F***	(5′-t*a*ta***t***atataa***t***attata***t***atatatct-3′-)$_3$-Ψ-O-$(CH_2)_6$-S-S-$(CH_2)_6$-<br>Op-3′-CAACCAACCA*C*A-5′	34162.8	34149.2

[1] ESI MS. Notation: lowercase letters denote deoxyribonucleotides, uppercase letters denote ribonucleotides, 2′-O-methylribonucleotides are underlined; p – phosphodiester group; * – phosphorothioate group; Y – symmetric doubler residue; Ψ – trebler residue; ***t*** – *N*[3]-NPOM-dT.

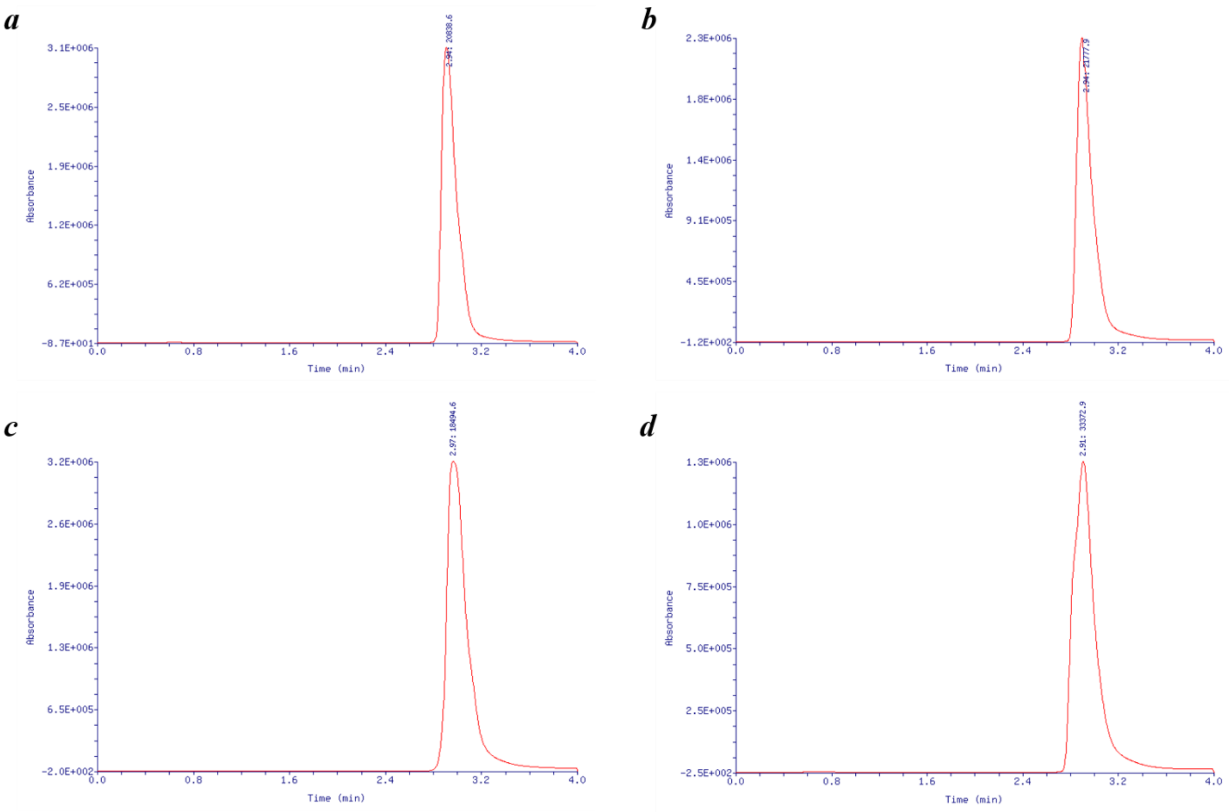

Figure S1. Elution profiles of LC-MS analysis of oligonucleotides: *a*) **A**, *b*) **B**, *c*) **C**, and *d*) **F** (Table S1).

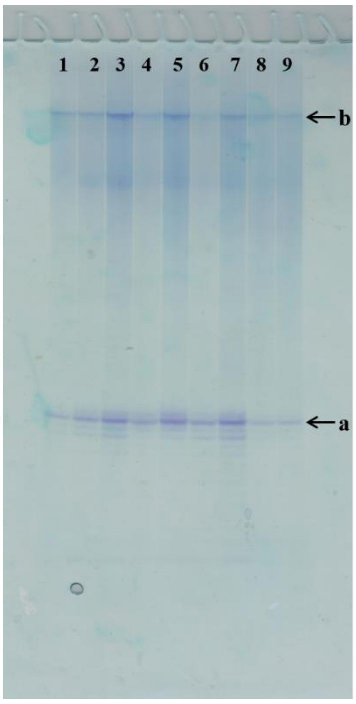

Figure S2. A 15% denaturing PAGE analysis of reaction mixtures from different syntheses of the starlet oligonucleotide. Lanes 1-9: different syntheses of the starlet; bands: *a* – truncated by-product related to incomplete C6 S-S modifier coupling; *b* – a band corresponding to full-length product that was excised in the preparative gel to isolate the starlet oligonucleotide. Conditions: 15% gel, 8 M urea, 1×Tris-borate buffer, pH 8.3, visualized by staining with Toluidine Blue.

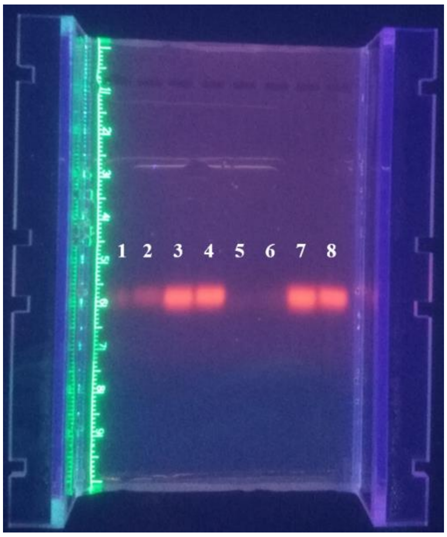

Figure S3. Control of template-assisted assembly of the C_{24} fullerene-like DNA shell by native electrophoresis in 0.75% agarose gel in 1×TAE buffer. Lanes 1 and 5 correspond to samples containing the dendron **D** before UV irradiation. Lanes 2 and 6 correspond to samples containing the 24-valent template **E** after UV irradiation. Lanes 3 and 7 correspond to the samples containing a complex of the template **E** and the C_{24} fullerene-like DNA shell from the starlet **F**. Lanes 4 and 8 correspond to samples containing a mixture of the template **E** and the starlet **F*** (Table S1). In the case of samples 1, 2, 3, 4, the incubation was carried out in a dry thermostat; in the case of samples 5, 6, 7, 8, the incubation was in a water bath. The gel was stained with ethidium bromide and visualized under UV light at 365 nm on a transilluminator.

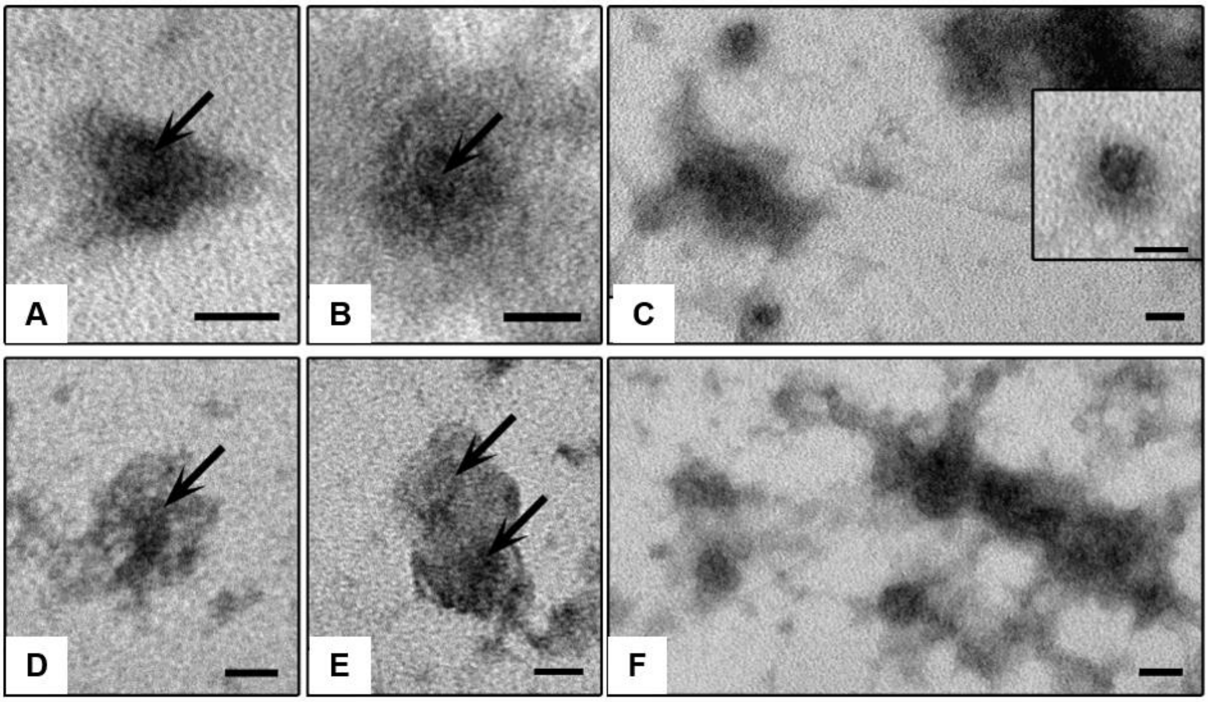

Figure S4. Representative images illustrating the attempted template-assisted assembly with control non-complementary starlet **F*** (Table S1) from the experiments on a water bath (A, B) or in a solid-state thermostat (D, E). Particles corresponding to the isolated templates: C - preparation obtained on a water bath; F - in a dry thermostat. The black arrows indicate the templates. TEM, negative staining with 1% uranyl acetate. Scale bar 25 nm.
